# Migration of Th1 Lymphocytes Is Regulated by CD152 (CTLA-4)-Mediated Signaling via PI3 Kinase-Dependent Akt Activation

**DOI:** 10.1371/journal.pone.0031391

**Published:** 2012-03-06

**Authors:** Karin Knieke, Holger Lingel, Kathrin Chamaon, Monika C. Brunner-Weinzierl

**Affiliations:** Department of Experimental Pediatrics, University Hospital Magdeburg, Otto von Guericke University Magdeburg, Magdeburg, Germany; La Jolla Institute for Allergy and Immunology, United States of America

## Abstract

Efficient adaptive immune responses require the localization of T lymphocytes in secondary lymphoid organs and inflamed tissues. To achieve correct localization of T lymphocytes, the migration of these cells is initiated and directed by adhesion molecules and chemokines. It has recently been shown that the inhibitory surface molecule CD152 (CTLA-4) initiates Th cell migration, but the molecular mechanism underlying this effect remains to be elucidated. Using CD4 T lymphocytes derived from OVA-specific TCR transgenic CD152-deficient and CD152-competent mice, we demonstrate that chemokine-triggered signal transduction is differentially regulated by CD152 via phosphoinositide 3-kinase (PI3K)-dependent activation of protein kinase B (PKB/Akt). In the presence of CD152 signaling, the chemoattractant CCL4 selectively induces the full activation of Akt via phosphorylation at threonine 308 and serine 473 in pro-inflammatory Th lymphocytes expressing the cognate chemokine receptor CCR5. Akt signals lead to cytoskeleton rearrangements, which are indispensable for migration. Therefore, this novel Akt-modulating function of CD152 signals affecting T cell migration demonstrates that boosting CD152 or its down-stream signal transduction could aid therapies aimed at sensitizing T lymphocytes for optimal migration, thus contributing to a precise and effective immune response.

## Introduction

Migration of immune cells, such as T lymphocytes, is a prerequisite for achieving adaptive immune responses. The recirculation of T lymphocytes and their localization at sites of antigenic challenge are regulated by the interaction of chemokines (CCL, CXCL) with cognate chemokine receptors (CCR, CXCR) and subsequently activated adhesion molecules. The expression of diverse chemokine receptors characterizes different subsets of T lymphocytes and drives their localization to specific tissues (reviewed in [1]). For example, recirculating naïve T lymphocytes are directed via the homeostatic CCR7 receptor to lymphoid organs [2], [3], whereas pro-inflammatory Th1 lymphocytes upregulate the expression of CXCR3 and CCR5 [4] and are thereby guided to inflamed tissues.

Chemokine receptors are G-protein-coupled receptors (GPCR), which can be rapidly desensitized by G-protein-coupled kinases (GRK) after agonist activation, resulting in limited migration capacities [5], [6]. Dissociating G-protein subunits can in turn activate signaling cascades, including those involving protein kinase C (PKC), mitogen-activated protein kinase (MAPK) and PI3K. PI3K is known to regulate the migration of T lymphocytes and ultimately leads to reorganization of the cytoskeleton, which is necessary to achieve anterior-posterior orientation of immune cells and for site-directed motility. Several target molecules of PI3K have been shown to be involved in chemotactic processes, such as Tec - Kinases, GTPases and Akt [7], [8], [9], [10], [11], [12]. The small GTPase Rac1 controls actin dynamics and membrane protrusions [13]. Active Akt is recruited to the leading edge of cells and is mainly involved in the development of cell orientation via the reorganization of actin filaments.

Costimulation is required by T lymphocytes to achieve full activation, which is associated with proliferation, differentiation, survival and migration. The main positive costimulator of these cells is the constitutively expressed Ig family member CD28, whereas its homologue CD152 is often reported to act as a negative regulator of T cell activation [14], [15], [16]. Both molecules bind to the B7 family members CD80 and CD86, with CD152 displaying a higher affinity and being upregulated upon T cell activation. CD152 signaling in activated T lymphocytes, where the CD152 pathway exerts its main function, has been shown to induce cell cycle arrest, reduced cytokine production, survival and migration. Signal transduction by CD152 is not well understood; it has been shown that this molecule binds PI3K, Src homology 2 (SH2) domain-containing protein tyrosine phosphatase 2 (SHP-2) and serine/threonine phosphatase 2A (PP2A) and mediates the inactivation of pro-apoptotic molecules, such as Bad (Bcl-2-antagonist of cell death) [14], [15], [17]. With respect to the central signaling kinase Akt, CD152 signaling mediates Akt activation on Th1 and Th2 lymphocytes, which in turn inactivates the Fas ligand transcription factor FOXO3 [18]. In addition, the long-lived CD28^null^ population has been shown to maintain resistance to apoptosis via the activation of Akt [19]. However, survival only requires a low level of Akt phosphorylation in T lymphocytes [20], and it is not known whether CD152 induces strong Akt activation in T lymphocytes, which is necessary for migration.

The costimulatory molecules CD28 and CD152 both increase integrin-mediated adhesion processes via β1-integrin receptors and LFA-1 integrin and enhance chemokine-driven direction of T lymphocytes into and within secondary lymphoid organs and inflamed tissues [21], [22], [23], [24]. Although the increase of Th1 cell migration in the presence of CD152 signals is based on increased expression of the chemokine receptors CCR5 and CCR7 [24], it is likely that this is not the only mechanism mediated by CD152 to drive migration. In this study, we show that Th1 lymphocytes presenting similar levels of CCR5 and CCR7 expression exhibit considerably improved migration when they receive a CD152 signal. Therefore, we hypothesized that intracellular signal transduction via CCR5 in Th1 lymphocytes is controlled by CD152 interference with CCR5 signaling, such as through desensitization of CCR5, CCR5-mediated adhesion, CCR5-mediated reorganization of the cytoskeleton and/or CCR5-mediated dissociation of G-protein subunits that activate signaling cascades, such as the MAPK and protein kinase C (PKC) pathways. To analyze the signaling axis responsible for this improved migration, we assayed key signal transduction molecules involved in different chemokine receptor axes, including GRK2, Erk, Rac, PKCθ, PI3K, and Akt, in the absence or presence of CD152 signaling via biochemical analysis and subsequent functional analysis.

## Materials and Methods

### Mice

CD152-competent (CD152^+/+^) and CD152-deficient (CD152^−/−^) OTII mice transgenic (tg) for the OVA-specific TCR^tg/tg^ in a C57BL/6 background and C57BL/6 mice were bred under specific pathogen-free conditions in the animal facility at the Federal Institue of Risk Assessment Bundesinstitut für Risikobewertung (Berlin) and the animal facility at the Otto-von-Guericke University Magdeburg and were used at 5 to 10 weeks of age.

### Antibodies, cytokines and reagents

The following antibodies against murine antigens were used in PE- and APC-conjugated forms: anti-CCR5 (C34-3448), PE-conjugated rat IgG_2_C (BD Biosciences, Franklin Lakes, USA), anti-CCR7 (4B12) and PE- and APC-conjugated ratIgG_2_a (eBioscience, San Diego, USA). Anti-IL-4 (11B11) and anti-CD4 (GK-1.5/4) were purified from hybridoma supernatants using protein G and were controlled by HPLC and FACS analysis. Recombinant chemokines and recombinant IL-12 were purchased from R&D Systems (Minneapolis, USA). For isolation of lymphocytes, magnetic microbeads, anti-CD4, anti-CD62L, anti-CD90, and anti-FITC multisort microbeads were purchased from Miltenyi Biotech (Bergisch Gladbach, Germany). The PI3 kinase inhibitors wortmannin and Ly294.002 were obtained from Sigma Aldrich (St. Louis, USA). Akt inhibitor II (SH-5) and the PKCθ inhibitor Rottlerin were purchased from Calbiochem (Darmstadt, Germany).

### Cell isolation and stimulation

Isolation of naïve CD62L^high^CD4^+^ T lymphocytes from CD152-competent and CD152-deficient OTII mice was performed using magnetic cell separation (MACS™, Miltenyi, Bergisch Gladbach, Germany). Isolated TCR^tg^ CD152-deficient CD4^+^ T lymphocytes were controlled to prevent them from undergoing spontaneous proliferation, as is observed in CD152 knockout CD4^+^ T lymphocytes (data not shown, reviewed in [16]). These cells were stimulated with 5 µg/ml OVA_323–339_ peptide (provided by Schneider-Mergener, Charité, Berlin, Germany) and using T cell-depleted splenocytes as APCs. On day 6 after the onset of stimulation, the recall response was initiated by adding freshly isolated APCs and OVA peptide to density gradient-purified cells. For Th1 polarization, recombinant IL-12 (10 ng/ml) and anti-IL-4 (6 µg/ml) were added to the cultures. The differentiation of TCR^tg^ CD152-competent or CD152-deficient Th1 lymphocytes was routinely controlled so that the cells showed similar expression of activation-associated molecules (CD25, CD44, CD62L^low^, IFNγ), as demonstrated previously (Fig. 7 in [24]).

### Chemotaxis assay

Th1 lymphocytes were collected on days 4–6 following the initiation of the recall response. After density centrifugation using Histopaque 1083 (Sigma Aldrich, St. Louis, USA), chemotaxis assays were performed using 5 µm pore-size polycarbonate Transwell filters (Corning Inc., Corning, USA). A total of 5×10^5^ cells in 100 µl of assay medium were added to fibronectin-coated filters (10 µg/ml, Invitrogen, Darmstadt, Germany), and a chemokine dilution (10 nM CCL4, 10 nM CCL19) or medium was added to the lower chambers. Migrated lymphocytes were recovered after 90 min of incubation at 37°C. The frequency of migrated lymphocytes was determined by staining with anti-CD4 (L3T4, BD Biosciences, Franklin Lakes, USA) and comparing the numbers to a fixed amount of fluorescent beads (Fluoresbrite microparticles 20 µm, Polysciences Inc., Warrington, USA) by flow cytometry. All determinations were performed in triplicate. The chemotactic index indicates the ratio of Th lymphocytes that migrated in response to chemokine versus medium treatment.

### Phospho-protein staining

Phosphorylated isoforms of ERK and Akt were detected by flow cytometry. T lymphocytes were starved in serum-free medium for 3 hours, and chemokine receptors were activated triggered by the addition of 50 nM CCL4 for the indicated times. The cells were immediately fixed in 2% paraformaldehyde and were permeabilized with 90% methanol prior to staining with phospho-specific Akt (pSerine 473 and pThreonine 308); pan-Akt antibodies (Cell Signaling Technology, Danvers, USA); or PE-conjugated phospho-specific Erk1/2 antibodies (BD Biosciences, Franklin Lakes, USA) for 1 h at room temperature (RT). Akt antibodies were detected using a goat anti-rabbit IgG-Alexa488-conjugated secondary F(ab)_2_-antibody (Cell Signaling Technology, Danvers, USA) following incubation for 30 min at RT.

### G-LISA and western blot analyses

G-LISA was performed according to the manufacturers' instructions (Cytoskeleton, Denver, USA). Briefly, on day 5 following initiation of the recall responses, T lymphocytes were starved in serum-free medium for 3 hours. Rac activation was induced in 2×10^6^ T lymphocytes by the application of 20 nM CCL4 for the indicated times. The cells were then immediately lysed and snap frozen. The protein concentrations of all samples were equalized, and 15 µg of protein (in 50 µl) was applied to a Rac-GTP-binding microplate in duplicate. Active Rac was identified by incubation with an anti-Rac 1,2,3-recognizing antibody followed by an HRP-coupled secondary antibody, and detection was performed at 490 nm using a TECAN-infinite M200-luminometer.

Gel electrophoresis and western blotting were performed as follows: 5×10^6^ lymphocytes were starved in serum-free medium for 3 hours prior to the addition of stimulating chemokines. The cells were placed on ice, washed in ice-cold PBS and lysed. The lysates were incubated with reducing Laemmli buffer for 5 min at 95°C, run on a 12.5% polyacrylamide gel, and transferred to a nitrocellulose membrane. The membrane was than incubated with anti-GRK2 (Y137, Abcam, Cambridge, UK), anti-phospho-GRK2 (pSerine S670, Abcam), or anti-α-tubulin (DM1A, Calbiochem, Darmstadt, Germany). HRP-coupled secondary antibodies (Abcam) were used for detection with ECL reagents (GE Healthcare, Buckinghamshire, UK). The band intensity of pGRK2 relative to GRK2 was quantified using Image Gauge 4.0 software (Fuji Photo Film, Tokyo, Japan).

### Statistical analysis

**Figure 1:**
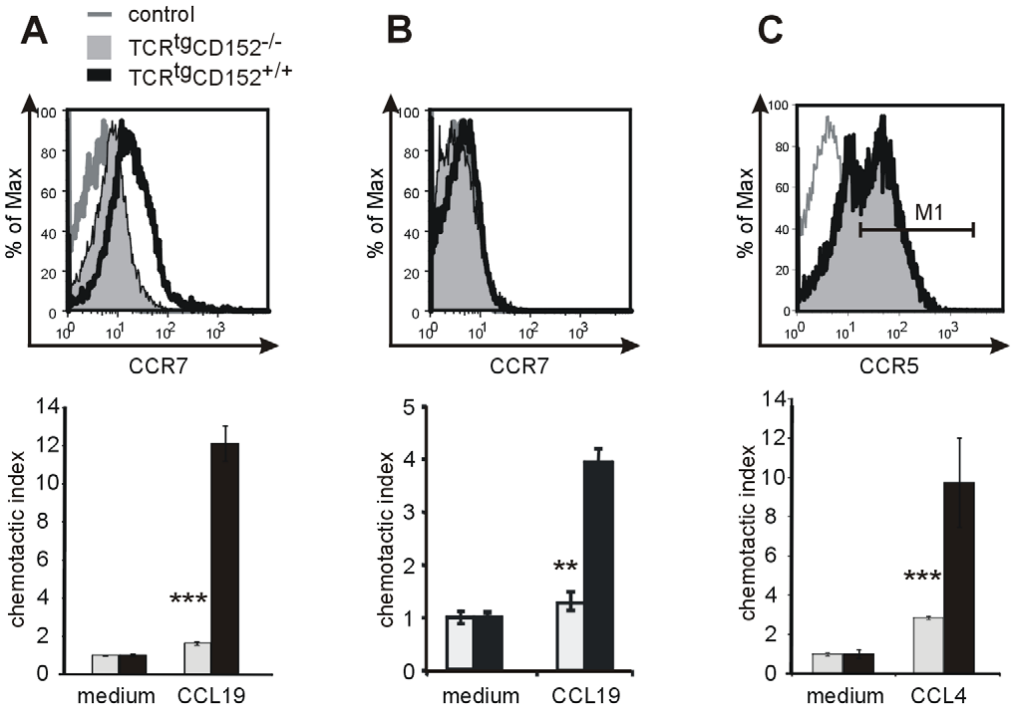
Chemokine receptor expression in activated T lymphocytes does not reflect the chemotactic capacities of corresponding chemokines. CD4^+^ T lymphocytes from TCR^tg^CD152-deficient (CD152^−/−^) and TCR^tg^CD152-competent (CD152^+/+^) mice were analyzed to detect chemokine receptor expression by flow cytometry (upper panels) and in chemotaxis assays (lower panels) on day 3 after the initiation of recall responses. **A** CCR7 expression and migration toward medium or CCL19 were detected in T lymphocytes. **B** Th1-differentiated CD4^+^ T lymphocytes were analyzed for CCR7 expression and migration behavior toward medium or CCL19. **C** Th1 lymphocytes exhibited similar CCR5 expression levels (M1: TCR^tg^CD152-deficient, 60%; TCR^tg^CD152-competent, 60%) but different migration rates in transwell systems. Representative data from two experiments are shown.

**Figure 2:**
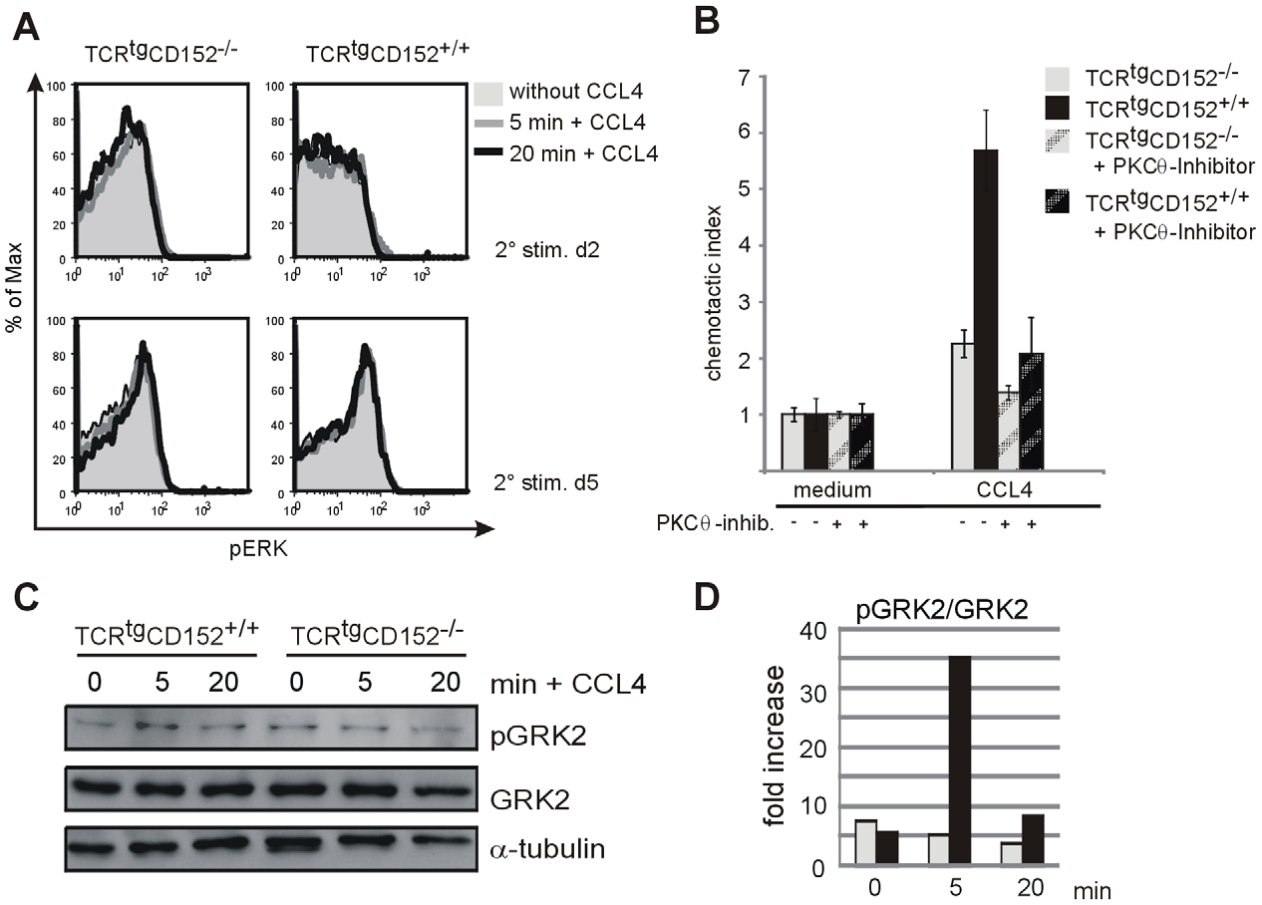
Effect of induction with the chemokine CCL4 on signaling molecules. **A** Th1 lymphocytes from TCR^tg^CD152-deficient and TCR^tg^CD152-competent mice were analyzed for ERK phosphorylation on day 2 and day 5 of a recall response. Signaling via CCR5 was induced in serum-starved lymphocytes by treatment with 20 nM CCL4 for 5 (heavy black line) or 20 min (heavy grey line). Filled histograms indicate ERK phosphorylation before CCL4 application. **B** Migration assay in Th1 lymphocytes from TCR^tg^CD152-deficient and TCR^tg^CD152-competent mice on day 5 of a recall response. A portion of the lymphocytes was incubated with the PKCθ-specific inhibitor Rottlerin for 20 h. **C** Western blot detection of signaling proteins in lysates of TCR^tg^CD152-deficient and TCR^tg^CD152-competent Th1 lymphocytes on day 5 of a recall response. The cells were treated as described in Fig. 2 A. **D** The band intensity of pGRK2 relative to GRK2 was quantified using Image Gauge 4.0. Representative data from at least two experiments are shown.

**Figure 3:**
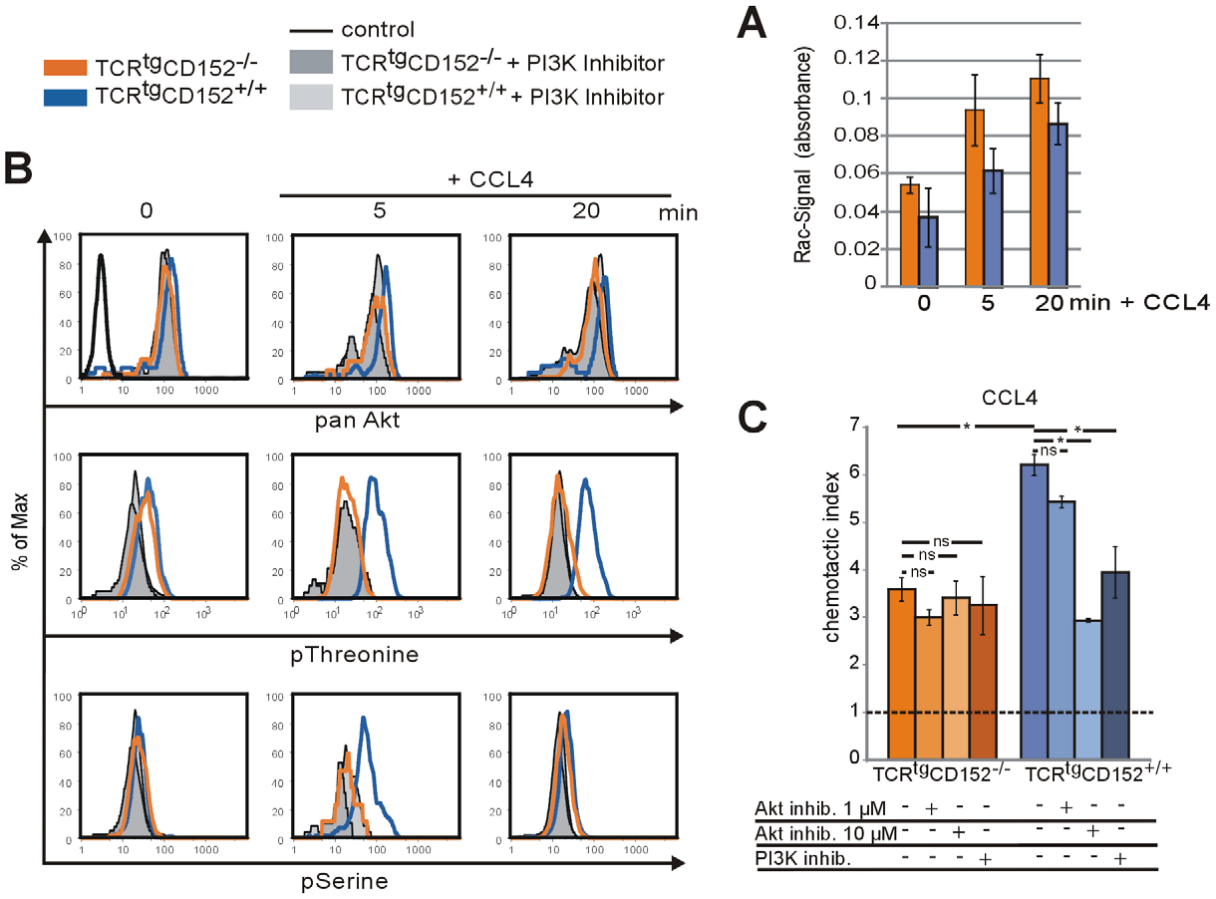
Chemokine receptor signaling affects signaling molecules involved in cytoskeleton rearrangements. **A** Activated Rac was detected by G-LISA in Th1 lymphocytes from TCR^tg^CD152-deficient and TCR^tg^CD152-competent mice on day 5 of a recall response. Rac activation was induced in serum-starved lymphocytes by treatment with 20 nM CCL4 for 5 or 20 min. **B** Th1 lymphocytes from TCR^tg^CD152-deficient and TCR^tg^CD152-competent mice on day 5 of a recall response were incubated with or without 20 nM CCL4 for 5 or 20 min. Fixed and permeabilized lymphocytes were analyzed by flow cytometry for Akt activation using antibodies specific for phosphorylated Akt or total Akt. A protion of the lymphocyteswere incubated for 20 h with the PI3K inhibitor Ly294.002 (filled histograms). Histograms show the expression of Akt on T lymphocytes. **C** Th1 lymphocytes from TCR^tg^CD152-deficient and TCR^tg^CD152-competent mice were analyzed to determine their migration affinities for the inflammatory chemokine CCL4 in a transwell system on day 5 of a recall response. A portion of the Th1 lymphocytes were incubated for 20 h with a PI3K inhibitor or Akt inhibitor II, as indicated. The dotted line indicates basal migration toward medium (ns, not significant). Representative data from at least two experiments are shown.

Statistical analysis was performed using a two-tailed unpaired Student's t-test (Figures 1, 2, 3), and p<0.05 (*), p<0.01 (**), and p<0.001 (***) were considered to represent significant differences.

## Results

### Chemokine receptor expression provides limited information regarding CD152-mediated chemotactic capacity

The expression of chemokine receptors on the surface of immune reactive cells has often been used to predict migratory behavior. To investigate if this is also the case for CD152-mediated migration [24], we stimulated CD4^+^ T lymphocytes from OTII^tg^CD152-deficient and OTII^tg^CD152-competent mice with OVA peptide and used CD90-depleted splenocytes as APCs, with some of these experiments being performed under Th1 conditions, as indicated. The cells were used 3 days after the initiation of a recall response for detection of chemokine receptors or for functional migration assays in transwell systems. We determined that CCR7 expression was correlated with the chemotactic response to the corresponding chemokine, CCL19 (Fig. 1 A). CD152-deficient T lymphocytes showed low/no CCR7 expression, and only a few CD4^+^ T lymphocytes migrated to CCL19. Because the detection limit of the molecules commonly used in the procedures for performing cytometric measurements is approximately 2,000 molecules per cell [25], these lymphocytes might still express CCR7 at low levels that are sufficient to stimulate them to migrate to CCL19. Accordingly, the elevated CCR7 expression in CD152-competent Th lymphocytes resulted in 12-fold greater attraction of Th lymphocytes to CCL19 than to the medium. Following generation of Th1 lymphocytes by the addition of recombinant IL-12 and blocking IL-4, almost no CCR7 expression was detected in CD152-deficient and CD152-competent T lymphocytes (Fig. 1 B, top panel). Nevertheless, an improved chemotactic capacity was demonstrated in CD152-competent CD4^+^ T lymphocytes. The chemotactic index of CD152-deficient lymphocytes in response to CCL19 remained by 1.2 whereas 4 times more CD152-competent T lymphocytes migrated to CCL19 than to the medium control (Fig. 1 B, lower panel). We have recently shown that the higher expression of the pro-inflammatory chemokine receptor CCR5 on CD152-competent Th1 lymphocytes compared to CD152-deficient Th1 lymphocytes is correlated with an improved chemotactic response to the binding of chemokine CCL4 on day 5 of a recall response [24]. Analyzing the expression and migratory behavior on day 3 of a recall response, when Th lymphocytes are still in an activated state, revealed similar expression of CCR5 on 60% of both TCR^tg^CD152-deficient and TCR^tg^CD152-competent Th1 lymphocytes (Fig. 1 C, top panel). However, CD152-deficient Th1 lymphocytes exhibited 2.8 times more migration to the chemoattractant CCL4 than to the medium at this time point (Fig. 1 C, lower panel), whereas CD152-competent Th1 lymphocytes presented a chemotactic index close to 10, representing a 3-fold higher migration rate than CD152-deficient Th1 lymphocytes (Fig. 1 C, lower panel). Thus, expression of the chemokine receptor CCR5 on TCR^tg^CD152-deficient and TCR^tg^CD152-competent Th1 lymphocytes does not explain the migration capacities of T lymphocytes, and CD152 signals likely affect chemokine receptor functionality.

### CD152 controls the specific migration of T lymphocytes by affecting signal transduction via CCR5

Despite similar expression of chemokine receptors, the enhanced specific migration of CD152-competent lymphocytes points toward a CD152-mediated mechanism altering intracellular signal transduction via CCR5. To monitor the CD152-controlled signal transduction pathways associated with CCR5, we now have an available system involving activated T-cells (+/− CD152 engagement) showing equal expression of chemokine receptors (Fig. 1). Using this system, we examined several typical signal transduction molecules induced by chemokine binding to CCR5, which is responsible for mediating the desensitization of CCR5 and reorganization of the cytoskeleton and may indicate dissociation of the G-protein subunits of CCR5, to identify the molecules responsible for the CD152-mediated increase in the directed migration of Th1 lymphocytes (Fig. 2, 3). We generated Th1 lymphocytes from TCR^tg^CD152-deficient and TCR^tg^CD152-competent mice. On day 2 and day 5 after the initiation of the recall response, the lymphocytes were starved in serum-free medium for 3 hours prior to the induction of chemokine receptor signaling via the addition of the chemokine CCL4 for the indicated times. Activation of the MAPK pathway was assessed by analyzing the phosphorylated, active isoform of ERK by flow cytometry (Fig. 2 A). No effect was detected following triggering with CCL4 for 5 min or 20 min compared to the ERK phosphorylation of Th1 lymphocytes during a recall response without the addition of a chemokine, independent of the presence of CD152 signals. Instead, the signaling molecule PKCθ, which has been shown to control chemokine receptor signaling in T lymphocytes [26], appears to be involved in the CD152-mediated migration of Th1 lymphocytes (Fig. 2 B). Lysates of TCR^tg^CD152-deficient and TCR^tg^CD152-competent Th1 lymphocytes were tested to detect the phosphorylated isoform of PKCθ after chemokine receptor triggering via the addition of CCL4. Treatment with CCL4 for 5 min led to a 20% increase in the level of active PKCθ in CD152-deficient CD4 T lymphocytes, compared with a 40% enhancement of PKCθ activation in the presence of CD152 signals (data not shown). PKCθ-dependent migration following CD152 signaling was tested functionally using the PKC inhibitor Rottlerin, which is specific for the PKCθ isoform. The specific migration of CD152-deficient Th1 lymphocytes, measured based on the chemotactic index, was inhibited by approximately 30% in the presence of Rottlerin, whereas the chemotactic index of CD152-competent Th1 lymphocytes was reduced twofold (63%) (Fig. 2 B).

Next, we analyzed the role of CD152 signaling during the desensitization of CCR5, which mediates the rapid modulation of migration capacities [27]. To determine the axis responsible for signaling via G-protein-coupled kinases (GRKs), we analyzed the typical signal transduction molecule GRK2. GRK2 is a suitable candidate for investigating the differential migration of T lymphocytes because ligand-bound receptors may vary in their functionality due to binding antagonistic chemokines or via phosphorylation through GRKs, especially GRK2, and the sequential binding of β-arrestins [27]. We analyzed TCR^tg^ Th1 lymphocytes in the absence or presence of CD152 signals to detect GRK2 and its phosphorylated, catalytically inactive isoform on day 5 following the initiation of a recall response (Fig. 2 C). Chemokine receptor signals were induced by CCL4 treatment for the indicated times, and protein lysates were tested to detect GRK2 isoforms using western blotting. GRK2 remained unchanged following CCL4 induction in TCR^tg^CD152-deficient and TCR^tg^CD152-competent Th1 lymphocytes when compared with the α-tubulin control. However, inactive GRK2 (pGRK2) was found to be selectively increased in TCR^tg^CD152-competent Th1 lymphocytes after chemokine induction for 5 min, and a 35-fold increase in the pGRK2/GRK2 ratio was observed, which permits prolonged signal transduction via the chemokine receptor (Fig. 2 C, D).

### CD152 signal transduction activates Akt

Next, we analyzed whether CD152 signaling interferes with the signaling axis involved in the chemokine-dependent trafficking of T lymphocytes from peripheral blood vessels to tissues [28]. For this purpose, we investigated the key signal transduction molecule PI3K and its downstream target Rac. Rac is a small GTPase that is a key player in cell spreading, polarization and leading edge formation [15], [29]. We detected the activity of Rac using G-LISA assays in lysates of TCR^tg^CD152-deficient and TCR^tg^CD152-competent Th1 lymphocytes on day 5 following the initiation of a recall response. We induced CCR5 chemokine receptor signaling in Th1 lymphocytes of TCR^tg^CD152-deficient and TCR^tg^CD152-competent mice via the application of CCL4 for 5 or 20 min (Fig. 3 A). The increase in Rac activation was similar over the examined time periods and did not depend on CD152 signaling (Fig. 3 A). The activation of another main target of PI3K, Akt, was also analyzed in Th1 lymphocytes via flow cytometry on day 3 (data not shown) and day 5 after the initiation of a recall response (Fig. 3 B). The total amount of Akt was comparable in CD152-deficient and CD152-competent Th1 lymphocytes and was unchanged in the presence of a PI3K inhibitor (Fig. 3 B, upper panel). In contrast, using phospho-specific antibodies for cytometric analyses, we determined that Akt activation was induced only in the presence of CD152 signals. Thr308 is the first site to be phosphorylated during the activation of Akt [30]. PI3K-dependent Thr308 phosphorylation occurred within 5 min after the induction of chemokine receptor signaling via the addition of CCL4 (Fig. 3 B, middle panels). The phosphorylation of Thr308 in Th1 lymphocytes from TCR^tg^CD152-competent mice remained stable for at least 20 min. In addition, the sequential phosphorylation of Akt at Ser670 was not detectable in Th1 lymphocytes from TCR^tg^CD152-deficient mice, although it was transiently up-regulated 5 min after the addition of CCL4 in CD152-competent CD4^+^ Th1 lymphocytes (Fig. 3 B, lower panels).

Moreover, functional chemotaxis assays supported the relevance of Akt activation with respect to the chemokine-induced migration of TCR^tg^CD152-competent Th1 lymphocytes (Fig. 3 C). Chemotaxis assays revealed that the chemotactic indices of CD152-deficient Th1 lymphocytes were not reduced significantly in the presence of the Akt inhibitor SH-5 or a PI3K inhibitor, whereas treatment with either the PI3K inhibitor or the Akt inhibitor decreased the migratory capacity of CD152-competent Th1 lymphocytes in a dose-dependent manner by up to 50% compared to Th1 lymphocytes not treated with inhibitors. Thus, our data unambiguously show that the central mechanism underlying CD152-induced migration is activation of the PI3K-Akt pathway.

**Figure 4:**
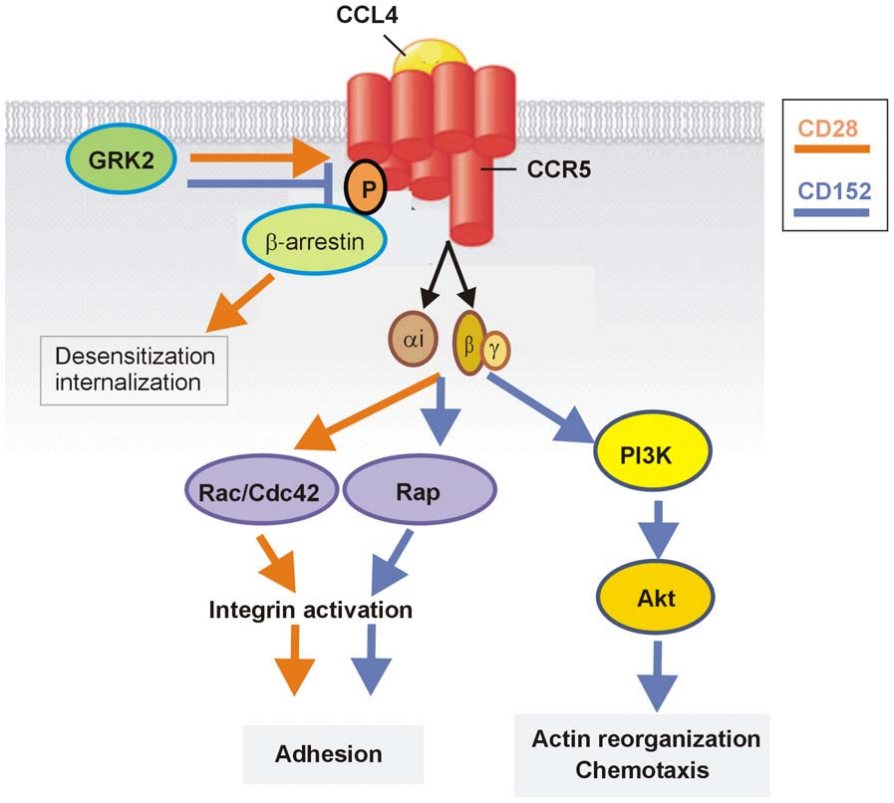
Role of CD28 and CD152 signals in signal transduction via the chemokine receptor CCR5 in Th1 lymphocytes. Upon binding of its ligand, CCL4, the CCR5 receptor is phosphorylated by CD28-induced GRK2. β-arrestins can now bind to CCR5 and initiate desensitization, which contributes to the degradation or recycling of CCR5. CD152 engagement leads to the inactivation of GRK2, and the phosphorylation of CCR5 is prevented. CD28 and CD152 signal-induced activation of integrins by the Gβγ subunit and via the GTPases Rac1 and Cdc42 or Rap1 ultimately leads to lymphocyte adhesion. Chemokine-induced activation of PI3K and the subsequent phosphorylation and activation of Akt are only initiated in the presence of CD152 signaling, and it is only under these conditions that specific migration occurs along chemokine gradients (orange arrows indicate signal transduction under CD28 signaling, and blue arrows indicate signal transduction under CD152 signaling). The figure shows only those signaling pathways controlled by CD152.

This study shows that Th1 lymphocytes presenting similar levels of CCR5 and CCR7 expression exhibit improved migration following receiving a CD152 signal. Using biochemical and functional assays, we show that CD152-mediated control of specific migration is at least partially mediated by CD152 inhibition of GRK2, although this process is mainly regulated by the full activation of Akt, and both mechanisms together enhance the capacity of Th1 lymphocytes to direct migration toward inflammatory CCL4. Thus, our data show that CD152 controls at least two signaling axes associated with CCR5 (Fig. 4).

## Discussion

CD152 signals enhance the motility [23] and the chemotaxis of Th1 lymphocytes [24]. The results of this study point to a mechanism underlying the increased directed migration of Th1 lymphocytes mediated by CD152 signals based on prolonged chemokine receptor signaling and PI3K-dependent Akt activation.

Our results clearly demonstrate that the expression of chemokine receptors reveals only limited information regarding the migratory capacities of T lymphocytes and indicate the usefulness of monitoring via functional migratory assays (Fig. 1). Differential migratory affinities toward CXCL12 have been described in B lymphocytes. However, the single chemokine receptor CXCR4 is present at all stages of B cell development [31]. Furthermore, it has been shown that the chemokine receptor CCR7, which is assumed to be down-regulated due to differentiation of Th1 or Th2 lymphocytes [32], directs Th1 lymphocytes toward its ligands CCL19 and CCL21 in functional assays [33]. In our system and others, the low levels of expression and rapid recycling of chemokine receptors may explain the restricted detection of their surface expression.

A characteristic molecule that induces cytoskeleton rearrangements is the small GTPase Rac. Rac, together with the GTPase Cdc42, organizes actin polymerization at the leading edge of cells, which is necessary for motility (reviewed in [34]). However, using G-LISA assays, we did not detect significant differences in Rac activation in CD152-deficient or CD152-competent Th1 lymphocytes following triggering with the CCR5 ligand CCL4 (Fig. 3 A). Moreover, recent studies have shown that T cell motility can be maintained, independent of Rac activation and F-actin polymerization by L-plastin, via regulation of the stability and the polarization of F-actin and CCR7 [35]. Whether Rac activation is initiated in CD152-competent and CD152-deficient Th1 lymphocytes by the same signaling pathways involved in the activation of chemokine receptors, such as PI3K and Akt, remains to be determined. CD152-deficient lymphocytes may differentially activate Rac via integrin adhesion signals, whereas CD152-competent lymphocyte chemotactic signals likely involve Akt and Rac activation [34].

Although we could not detect a reduction in the total amount of GRK2 in TCR^tg^CD152-competent Th1 lymphocytes compared to TCR^tg^CD152-deficient Th1 lymphocytes (Fig. 2 C), chemokine receptor signaling is likely dependent on GRK2 [27]. Because GRK2 is a ubiquitous molecule, the generally high levels of GRK2 in T lymphocytes [36] may obscure any chemokine-induced changes in total GRK2 in our system. Indeed, chemokine-induced migration of T lymphocytes has been shown to be attenuated by GRK2 (reviewed in [27], [37]). In accordance with our findings, decreased levels of GRK2 expression in GRK2^+/−^ mice were previously proposed to enhance migration toward the CCR5 ligand CCL4 [38]. However, the catalytically inactive isoform of GRK2, which is phosphorylated at serine 670 [6], was found to be present in increased levels in TCR^tg^CD152-competent Th1 lymphocytes, leading to decreased desensitization of the chemokine receptor by β-arrestins, which bind to phosphorylated receptors. The reassociated G-proteins recouple with the chemokine receptor, allowing prolonged signal transduction via CCR5 expressed on the cell surface (Fig. 2 C, D). Additionally, other desensitization mechanisms not examined in the present study may be involved, as it has been reported for CCR5 that ligand-bound receptors can pass through endosomal recycling pathways without the dissociation of chemokines, following which functionally desensitized CCR5 is re-expressed at the cell surface [39].

It is unlikely that the differences in migration and chemokine-induced signal transduction in the presence and absence of CD152 are due to enhanced CD28 engagement, as this would be reflected in altered expression of activation-associated molecules, such as CD69, CD44, and CD25, which is not the case under the conditions employed in the present study [24]. Moreover, no differences were detected between CD152-deficient and CD152-competent Th1 lymphocytes concerning the activation of the signaling molecules ERK, GRK2, Akt or PKCθ prior to chemokine addition (Fig. 2 A, C and data not shown). The ERK pathway does not appear to be involved in CD152-mediated migration, as stimulation with CCL4 did not increase its activation level (Fig. 2 A). Although ERK activation can be induced by homeostatic CXCL12 in B lymphocytes [40] and CXCL8 in T cell lines [41], this may differ in the case of primary Th1 lymphocytes because signaling pathways vary in terms of the cell type, ligands, receptors and kinetics involved. Additionally, the diverse chemokine receptor profiles expressed by different T cell subpopulations, such as antigen-experienced resting T lymphocytes and naïve or recently activated T lymphocytes [32], most likely influence signaling pathways differently.

Akt was selectively activated by chemokine induction in CD152-competent Th1 lymphocytes (Fig. 3 B), and chemotaxis induced via CCR5 was markedly reduced in the presence of Akt or PI3K inhibitors (Fig. 3 C). These data correspond to the described roles of PI3K in lymphocyte migration of mediating gradient sensing and cytoskeleton rearrangements [28], [34], [42]. Recently, differences in the dependency of PI3K signals have been demonstrated showing that the migration of human Th2 lymphocytes in vitro is PI3K independent [43], [44]. In contrast, the migration of isolated human T lymphocytes toward CXCL4 ex vivo has been found to be PI3K dependent, although after two days of TCR stimulation, PI3K became irrelevant [45], and migration was regulated via PKCθ [26]. Our results reveal that the directed migration of resting Th1 lymphocytes toward CCL4 remains dependent on PI3K-mediated Akt signals (Fig. 3 B) and is influenced by PKCθ signals (Fig. 2 B). These inconsistent findings may be a result of the different natures of the analyzed chemokine receptors and the corresponding differences in their expression and, ultimately, signaling properties. It is likely that inflammatory chemokine receptors, such as CCR5, are superior to homeostatic chemokine receptors because an inflammatory milieu creates an abundance of chemokines and adhesion ligands, and inducible chemokine receptors are expressed at higher levels than homeostatic ones.

Based on data for the CD28 and CD152 receptors for CD80/CD86 ligands, we propose the following model for the regulation of CCR5-directed migration (Fig. 4). Upon binding of its ligand, CCL4, the CCR5 receptor is phosphorylated by CD28-induced GRK2. β-arrestins can now bind to CCR5 and initiate the desensitization process, which contributes to the degradation or recycling of CCR5. Our data show that CD152 engagement leads to the inactivation of GRK2, while the likely phosphorylation of CCR5 is delayed or prevented. CD28 and CD152 signal-induced activation of integrins via the Gβγ-subunit and the GTPases Rac1 and Cdc42 or Rap1 ultimately leads to adhesion of lymphocytes [23]. Chemokine-induced activation of PI3K and the subsequent phosphorylation and activation of Akt are only initiated in the presence of CD152 signaling, and it is only under these conditions that specific migration along chemokine occurs. These data together with the observations that (i) CD152 induces resistance against activation-induced cell death [18], [23]; (ii) CD152 mediates the differentiation of high-quality memory T cells [46]; and (iii) CD152 signals in activated T cells lead to reduced adhesion and increased motility, which result in truncated T cell-APC interactions within lymph nodes [47], led us to hypothesize that CD152 expression and signaling select high quality T cells out of a heterogeneous population of stimulated T cells to become chemotactic. An appropriately selected population with the ability to migrate is important for the development of an effective and appropriate adaptive immune response in the periphery. Thus, CD152 might represent a master switch that determines the fate of an effector T cell and, ultimately, the quality of the immune response.

Our data suggest that full activation of Akt, which is necessary for chemotactic processes [14] including the polarization of cells to form an anterior-posterior axis [48], [49] and the activation of actin [50] to occur, is induced by CD152 signaling. In the absence of CD152, the localization of Th1 lymphocytes would be dominated by integrin-mediated, non-directed adhesion. Within the complex chemokine receptor signaling pathways, CD152 signals could enhance the Akt-mediated polarization of T lymphocytes and allow directed migration to occur. Therefore, CD152 may represent a promising target molecule for manipulating the localization of T lymphocytes to lymph nodes or to inflamed tissues, thereby, modulating immune responses.
